# Selective bromodomain and extra-terminal bromodomain inhibitor inactivates macrophages and hepatic stellate cells to inhibit liver inflammation and fibrosis

**DOI:** 10.1080/21655979.2022.2066756

**Published:** 2022-05-01

**Authors:** Rong Fu, Shi-Jia Zu, Yan-Jun Liu, Jia-Cheng Li, Wen-Zhen Dang, Li-Ping Liao, Li-Ping Liu, Pan-Yu Chen, He-Ming Huang, Kang-Hui Wu, Bing Zhou, Qin Pan, Cheng Luo, Yuan-Yuan Zhang, Guang-Ming Li

**Affiliations:** a Department of Gastroenterology, Xinhua Hospital, School of Medicine, Shanghai Jiaotong University, Shanghai, Yangpu District, China; b State Key Laboratory of Drug Research, Shanghai Institute of Materia Medica, Chinese Academy of Sciences, Shanghai, Zuchongzhi District, Shanghai, China; c University of Chinese Academy of Sciences, Huairou District, Beijing, China; d School of Pharmaceutical Science and Technology, Hangzhou Institute for Advanced Study, University of Chinese Academy of SciencesCAS, Hangzhou, Zhejiang, China; e Research center, Zhoupu Hospital affiliated to Shanghai University of Medicine and Health Sciences, Shanghai, Zhouyuan District, China

**Keywords:** BET inhibitor, macrophage, hepatic stellate cell, inflammation, fibrosis

## Abstract

Liver fibrosis occurs following inflammation triggered by the integrated actions of activated liver-resident macrophages (Kupffer cells) and hepatic stellate cells (HSCs), and the multiplicity of these mechanisms complicates drug therapy. Here, we demonstrate that the selective bromodomain and extra-terminal (BET) bromodomain inhibitor compound38 can block both the Janus kinase-signal transducer and activator of transcription and mitogen-activated protein kinase signaling pathways in macrophages, which decreased their secretion of proinflammatory cytokines in a dose-dependent manner. The inactivation of macrophages attenuated lipopolysaccharide-induced injurious inflammation concurrent with a reduction in F4/80+ cells, proinflammatory cytokine levels, and neutrophil infiltration. Moreover, compound 38 inhibited the Wnt/β-catenin and transforming growth factor-beta/SMAD signaling pathways to abolish the activation of HSCs. *In vivo*, compound 38 significantly decreased the collagen deposition and fibrotic area of a CCl_4_-induced liver fibrosis model, and restored the deficiency of activated HSCs and the upregulation of liver inflammation. These results highlight the potential role of compound 38 in treating liver fibrosis considering its simultaneous inhibitory effects on liver inflammation and related fibrosis.

## Graphical abstract

**Figure:**
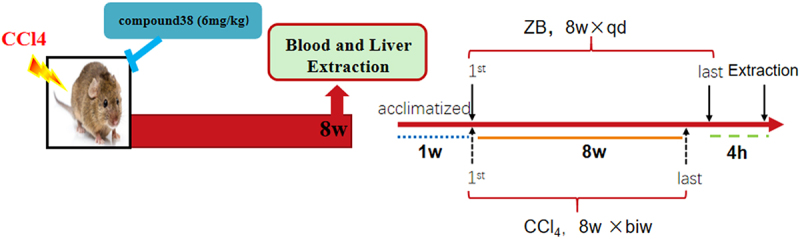

## Highlights

- Selective BET bromodomain inhibitor compound 38 exerts liver-protective effects
- Compound 38 inactivates Kupffer cells via inhibiting JAK-STAT and MAPK signaling
- Compound 38 inactivates of TGFβ/SMAD and Wnt/β-catenin signaling
- With anti-inflammatory and -fibrotic effects, Compound38 has clinical potential

## Introduction

The liver, as the central homeostatic organ in the human body, plays key roles in several essential physiological processes, including metabolism, detoxification, and immunity. Multiple etiologies (e.g., viral and microbial infection, alcoholic and lipo-oxidative toxicity, immune attack) induce liver injury and impair its functions [1–5]. Subsequently, this injury initiates lobular and/or portal inflammation based on Kupffer cell activation, cytokine release, and recruitment of inflammatory cells [6,7]. Liver inflammation, especially chronic inflammation, promotes the activation of hepatic stellate cells (HSCs), which are characterized by their fibroblast-like phenotype and excessive extracellular matrix (ECM) deposition, and induces liver fibrosis, which is the hallmark of liver cirrhosis, liver failure, or hepatocellular carcinoma [8–12]. However, the complicated mechanisms related to the multiple cell types underlying liver fibrosis have prevented development of an effective therapeutic intervention.

The bromodomain and extra-terminal (BET) family of chromatin proteins, including BRD2, BRD3, BRD4, and BRDT, serve as transcriptional cofactors to regulate transcription factor activity, exhibiting multiple biological functions via epigenetic regulatory mechanisms [13–17]. Recently, dysfunction of BET-containing proteins has been observed in various diseases [18–22], including inflammation [23,24], fibrotic disease [23,25,26], metabolic disorders and obesity [27], acute kidney injury [28], heart failure [29,30], pituitary adenomas [31], acute myeloid leukemia [32], breast cancer [33,34], and prostate cancer [35]. Thus, BET family proteins have emerged as promising drug targets for the treatment of cancer and inflammation. Moreover, several BET bromodomain inhibitors with different chemotypes have been discovered and advanced to clinical trials. Despite this progress, some BET inhibitors exhibited only a moderate selectively profile over non-BET bromodomains, which may lead to a potential safety issue. We previously identified a new fragment as a binder to the BET bromodomain, which was incorporated in the scaffold of ABBV-075, one of the most potent BET bromodomain inhibitors that is currently undergoing phase I clinical trials for the treatment of myelofibrosis. The derivative compound 38 demonstrated good properties with respect to druggability and oral pharmacokinetics, along with excellent affinity and selectivity binding to the BET bromodomain and good stability in microsomes [36].

Inflammation and fibrosis are inextricably linked during the process of liver disease, liver injury leading to inflammation, and the continuous liver inflammatory response promoting liver fibrosis [12]. Despite great progress in the study of liver fibrosis in recent years, there are still no approved drugs for its effective treatment. As a selective BET bromodomain inhibitor, we hypothesized that compound 38 was would facilitate the regulation of inflammation and fibrotic processes in liver.

To shed light on its pharmacological potential, in this study, compound 38 was employed in the treatment of lipopolysaccharide (LPS)/D-galactosamine-induced inflammation in HSCs and macrophages *in vitro* and in CCl_4_-induced liver fibrosis *in vivo* to identify its liver-specific targets. The molecular mechanisms underlying the effects of compound 38 were further investigated on the basis of RNA-sequencing (RNA-seq) and bioinformatics modeling. Overall, our study highlights compound 38 as a promising preclinical anti-inflammatory and anti-fibrotic candidate drug, which could exert a protective effect and impart benefits to patients with acute liver injury and liver fibrosis.

## Material and methods

### Cell culture

Raw264.7 murine macrophages, HSC-T6 rat HSCs, and LX2 human HSCs were incubated in high-glucose Dulbecco’s modified Eagle medium (DMEM) containing 1% (v/v) penicillin/streptomycin at 37°C with 5% CO_2_. The culture media of Raw264.7 and HSC-T6 cells were further supplemented with 10% fetal bovine serum (FBS; Gibco, Australia), whereas the culture medium of LX2 cells was supplemented with 15% FBS.

Primary bone marrow-derived macrophages (BMDMs) were obtained from the femur and tibia of C57BL/6 J male mice, aged 6–8 weeks. BMDMs were cultured in DMEM containing 1% antibiotics, 10% FBS, and 20 ng/mL macrophage-colony stimulating factor. The other culture conditions were as described above.

For treatment, both Raw264.7 and BMDM cells were co-incubated with 1 μg/mL LPS (Sigma, St. Louis, MO, USA) and compound 38 for 4 or 24 h. Adherent HSC-T6 and LX2 cells were starved in serum-free DMEM for 24 h, after which they were co-stimulated with 10 ng/mL recombinant human transforming growth factor (TGF)-β1 (PeproTech, China) and compound 38 (100 nM, 50 nM, 25 nM, 12.5 nM) for 24 h.

### Cell viability assay

Cells were plated at a density of 5000 cells per well with 100 μl of DMEM(contained with 10% FBS and 1% antibiotics). Cells were incubated at 37 C, 5% CO2 for 12 h before treating by different concentrations of compound 38. After compound 38 addition, cells were incubated for 24 h in same atmosphere. The viability study was conducted according the manufacturer’s instructions, using CellTiter-Glo 2.0 (Promega) [37].

### Animals

Adult male C57BL/6 J mice weighing 20–24 g were obtained from the Animal Center of the Chinese Academy of Medical Sciences. All animals were provided food and water *ad libitum* and housed under constant conditions at 21 ± 2°C with a 12-h light-dark cycle. All experimental operations were authorized by the Institute of Animal Care and Use Committee of Chinese Academy of Science, and performed at Shanghai Institute of Materia Medica. All animals were acclimatized to the new surroundings for one week after arriving at the animal center before starting the experiments.

### Establishment of the injury-induced acute inflammation model

LPS (Sigma; 2 mg/kg) and GalN (Sigma; 250 mg/kg) were mixed and dissolved in phosphate-buffered saline and administered to the mice by intraperitoneal (i.p.) injection [38]. Compound38 (6 mg/kg) was dissolved in 1% dimethyl sulfoxide (DMSO)/0.5% hydroxypropyl methyl cellulose (HPMC) and administered to the mice per os (p.o.) [36]. All mice were randomized into the normal control (NC; PBS, i.p.), model (LPS/GalN, i.p.), and Compound 38 (treated with Compound38, 6 mg/kg p.o., at 48 h, 24 h, and 2 h before receiving LPS/GalN, i.p.) groups. Mice in each group were exposed to solvent (1% DMSO and 0.5% HPMC, p.o.), with or without Compound38, at the volume of 10 μL/g. After 4-h treatment with LPS/GalN, all mice were sacrificed, and the blood and liver tissues were harvested. To evaluate the survival rate of LPS/GalN-treated animals administered Compound38, another experiment using the same protocol was performed for a period of one month.

### Establishment of the CCl_4_-induced liver fibrosis model

Compound 38 was dissolved in 1% DMSO/0.5% HPMC at a concentration of 6 mg/kg, and orally administered to the mice once a day five times a week; the solvent was treated at a dose of 10 µL/g. CCl_4_ was dissolved in olive oil and administered to the mice i.p. at 1 mL/kg twice a week for 8 weeks to induce liver fibrosis [39,40]. All mice were split into three groups randomly: control group administered 1% DMSO/0.5% HPMC (p.o.) and olive oil (i.p.), model group treated with CCl_4_ (i.p.) and 1% DMSO/0.5% HPMC (p.o.), and Compound38 group administered CCl_4_ (i.p.) and compound 38 (6 mg/kg, p.o.). At the end of the eighth week, the liver tissues were harvested to assess the fibrotic degree and blood samples were stored for subsequent analyses.

### Pathological assessment

Liver tissues were fixed in 4% paraformaldehyde for 48 h, and the tissue was embedded with paraffin and sectioned. Hematoxylin and eosin (H&E) and Masson staining was then performed according to standard procedures. Semi-quantitative analysis was performed using image processing software (Fiji Is Just Image J, NY, USA).

### Alanine aminotransferase (ALT) and aspartate aminotransferase (AST) activity

ALT and AST activities in the mouse serum were detected by a Hitachi 7020 automatic analyzer (Hitachi, Ltd., Japan), using the ALT assay kit (cat. no. C009-3-1) and AST assay kit (cat. no. C010-3-1) (Nanjing Jiancheng Bioengineering Institute, China), respectively.

### Enzyme-linked immunosorbent assay (ELISA)

The concentrations of interleukin (IL)-1β, tumor necrosis factor (TNF)-α, and IL-6 inflammatory cytokines in the LPS-stimulated cell culture supernatant were analyzed with respective ELISA kits (IL-1β, SN: 1,210,122; TNF-α, SN: 1,217,202; IL-6, SN: 1,210,602; DAKEWE, China), following the manufacturer instructions. Measurements were performed in triplicate for all samples.

### Reverse transcription-quantitative polymerase chain reaction (RT-qPCR)

Liver tissue and cell RNA were extracted using the FastPure Cell/Tissue Total RNA Isolation kit (Vazyme, China). The RNA was reverse-transcribed into cDNA following the instructions of HiScript III RT SuperMix for qPCR (+gDNA wiper) (Vazyme, China). PCR amplification was then performed using ChamQ SYBR qPCR Master Mix (Low ROX Premixed; Vazyme, China) on a Quant Studio 6 Flex Real-Time PCR system (ABI). The relative expression levels of target genes were determined using *Gapdh* for normalization with the 2^–∆∆Ct^ method. The primer sequences are given in Table S1.

### Western blotting

Total protein was extracted from the frozen liver tissue and cultured cells. After quantification with a bicinchoninic acid kit (Thermo Fisher Scientific, Waltham, MA, USA), the proteins were resolved by sodium dodecyl sulfate-polyacrylamide gel electrophoresis, subsequently transferred to nitrocellulose membranes, and incubated with primary antibodies at 4°C overnight. The membranes were then incubated with horseradish peroxidase-conjugated secondary antibodies at room temperature for at least 1 h. The target bands were visualized by an ECL imaging system (Clinx, ChemiScope 3400, China). Table S2 displays the details of the primary antibodies used in this study.

### Immunohistochemistry

After fixing with 4% paraformaldehyde for 48 h, the liver tissues were embedded with paraffin and sectioned, and then immunohistochemical staining was performed with antibodies against F4/80 (1:100, MAB5580, RD), alpha-smooth muscle actin (α-SMA; 1:100, GB13044), Ly-6 G (1:100; MAB1037-100, RD), and type I collagen (COL1A1; 1:100, GB11022-1). All steps were carried out according to the manufacturer instructions.

### Terminal deoxynucleotidyl transferase dUTP nick-end labeling (TUNEL)

One feature of apoptosis is DNA fragmentation, which can be be detected by TUNEL of DNA in dead cells.Liver tissues were fixed in 4% paraformaldehyde for 48 h prior to processing for paraffin embedding and sectioning. TUNEL staining was performed using a commercial kit (Roche, Mannheim, Germany) according to the manufacturer’s instructions.

### RNA-seq analysis

RNA samples, obtained from the mouse liver tissues and LX2 cells, were reverse-transcribed into cDNA and sequenced on the Illumina HiSeq 2000 platform. RNA sequences with an RNA integrity number ≥ 7.0 were filtered for quality control and used for analysis. STAR 2.5 was used to map the sequencing reads to the mm10 reference, and featurecounts software was used to quantify the gene expression [41,42]. The edgeR package was used to perform differential gene expression analysis [43]. The Benjamini and Hochberg method was used to adjust P-values, and the genes with significantly different expression levels were selected under a 5% false discovery rate cutoff value with a log2 fold-change > 1 for the LPS/GalN-exposed liver injury model and a threshold of fold change > 1.5 in the LX2 cells. The DAVID 6.8 bioinformatics platform and ClusterProfiler R package were further used to explore the biological functions associated with the differentially expressed genes [44]. Gene Set Enrichment Analysis (GSEA) was carried out with GSEA 4.1.0 software to investigate the variance in specific gene sets [45]. Network analysis was performed using STRING 11.0 and Cytoscape to determine the protein-protein interactions (PPIs) [46,47].

### Statistical analysis

All data are shown presented as the mean ± SD. GraphPad Prism 8.0 statistical software (GraphPad Software, Inc., La Jolla, CA, USA) was used to conduct the statistical analyses with a two-tailed unpaired test to compare different groups; P < 0.05 was considered significant.

## Results

### The selective BET bromodomain inhibitor compound 38 alleviated LPS-induced liver inflammation in vitro

#### Compound 38 exerted an anti-inflammatory effect

**Figure 1.**
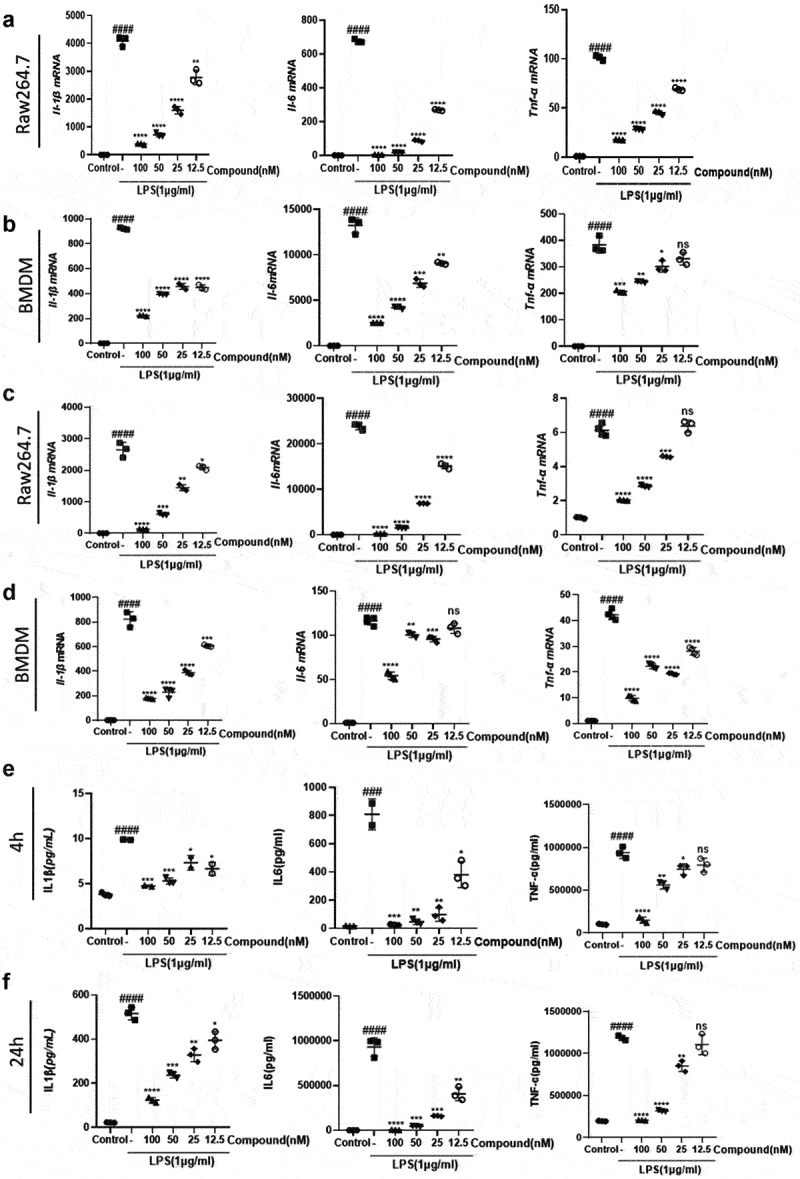
The selective BET inhibitor compound 38 inhibits the LPS-induced inflammatory response.

Hepatic macrophages secrete pro-inflammatory cytokines, recruiting additional immune cells to aggravate hepatic injury; thus, they have been regarded as potential targets for treating liver injury and liver fibrosis [11,48]. It is now recognized that anti-inflammatory effects are very important in combating liver injury. We found that the dramatic increase in the mRNA expression levels of pro-inflammatory factors (*Il-1β, Il-6*, and *Tnf-α*) after LPS stimulation of Raw264.7 and BMDM cells was greatly relieved after the administration of compound 38 in a dose-dependent manner (Figure 1a, b). Prolonged co-incubation up to 24 h yielded similar results (Figure 1c, d). ELISA further confirmed that compound 38 could greatly attenuate the induced secretion of inflammatory proteins (IL-1β, IL-6, and TNF-α) caused by LPS stimulation (Figure 1e, f). These findings demonstrated that compound 38 can exert a powerful anti-inflammatory effect *in vitro*. Kupffer cells are liver macrophages whose functions are highly specialized and different from those of other anatomical sites [49,50]. The primary kupffer cells were extracted, having the same treatment as Raw264.7 and BMDM cells. Finally, coming to the same conclusion that the expression level of *Il-1β, Il-6*, and *Tnf-α* mRNA was dramatic decrease by the administration of compound 38 in a dose-dependent manner Figure 7b. In addition, compound 38 had a negligible effect on cell viability Figure 7b.

(a, b) Four-hour administration with the selective BET inhibitor compound 38 downregulated the mRNA levels of *Il-1β, Il-6*, and *Tnf-α* in mouse Raw264.7 (a) and primary BMDM (b) cells induced by LPS. (c, d) Exposure for 24 h decreased the mRNA levels of *Il-1β, Il-6*, and *Tnf-α* in Raw264.7 (c) and BMDM (d) cells induced by LPS. (e, f) ELISA demonstrated the inhibitory effect of compound 38 on IL-1β, IL-6, and TNF-α expression of Raw264.7 cells after 4 h (e) and 24 h (f) exposure. Data represent the mean ± SD. *P < 0.05, **P < 0.01, ***P < 0.001, ****P < 0.0001 compared to the LPS-treated group. #P < 0.05, ##P < 0.01, ###P < 0.001, ####P < 0.0001 compared to the control group.

#### Compound 38 pretreatment attenuates LPS/GalN-induced liver injury

**Figure 2.**
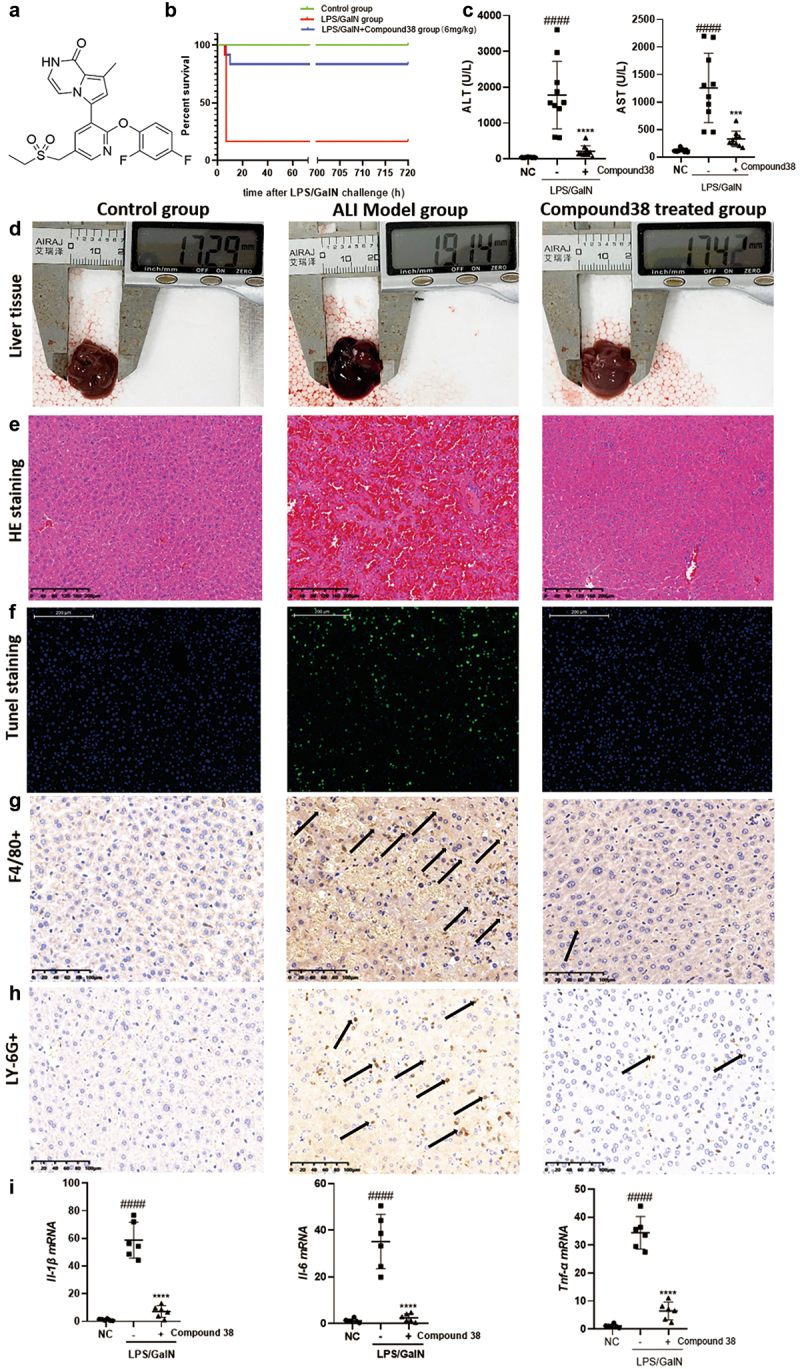
Compound 38 treatment attenuates inflammatory responses in the LPS/ GalN-induced acute liver injury (ALI) mouse model *in vivo.*

Compound 38 pretreatment (6 mg/kg) significantly reduced mortality and improved the survival rate of mice in the LPS/GalN liver injury model. Even when extending the observation time to one month, the survival state was found to be stable (Figure 2b). LPS/GalN injection caused severe hepatocyte damage and elevated serum transaminases (ALT and AST), which were reversed by compound 38 administration (Figure 2c). In terms of morphology, the inflammation and congestion in the model group were severe, but were relieved in the compound 38 pre-treated group (Figure 2 d). Histopathological examinations confirmed these effects. H&E staining showed that the liver cells in the model group were disordered, with significant red blood cell infiltration, and some liver cells were swollen, ruptured, and necrotic; however, the phenotype of the compound 38-administered group was similar to that of the blank control group (Figure 2e). TUNEL staining revealed a significant degree of hepatocyte necrosis in the livers of the LPS/GalN-stimulated group, which was greatly alleviated by compound 38 pre-treatment (Figure 2f).

(a) The chemical structure of compound 38. (b) Survival curves for mice in the normal control (NC), ALI model, and compound 38-treated groups (n = 12 per group). (c) Serum ALT and AST levels in mice from the NC, ALI model, and compound 38-treated groups (n = 10 per group). (d) Morphological changes in the livers of mice from the indicated groups. (e and f) Representative images of the histological examination of liver tissues. H&E (e) and TUNEL (f) staining of the indicated groups of mice. (g and h) Representative images of immunohistochemical staining of F4/80+ macrophages (g) and LY-6 G+ neutrophils (h) within the liver tissue. (i) *Il-1β, Il-6*, and *Tnf-α* mRNA levels in the liver tissues of different groups quantified by RT-qPCR analysis. Data represent the mean ± SD. *P < 0.05, **P < 0.01, ***P < 0.001, ****P < 0.0001 compared to LPS/GalN-stimulated mice. #P < 0.05, ##P < 0.01, ###P < 0.001, ####P < 0.0001 compared to the control group.

Kupffer cells, which are self-renewing resident liver macrophages, release inflammatory cytokines after activation by liver injury to boost monocytes infiltrating the liver, which produces numerous exogenous macrophages [51]. To further detect the inflammatory cell infiltration of the liver, immunohistochemistry staining was performed. The infiltration of macrophages (labeled by F4/80) and neutrophils (labeled by LY-6 G) was significantly decreased in the compound 38-treated group compared with that of the model group (Figure 2g, h). RT-qPCR further revealed that the expression of inflammation-related cytokines *Il-1β, Il-6*, and *Tnf-α* was markedly inhibited by compound 38 administration (Figure 2i). Overall, these findings demonstrated that compound 38 also exhibits a powerful anti-inflammatory effect *in vivo*.

### Compound 38 attenuated the proinflammatory phenotype of Kupffer cells by inhibiting inflammatory signaling pathways

**Figure 3.**
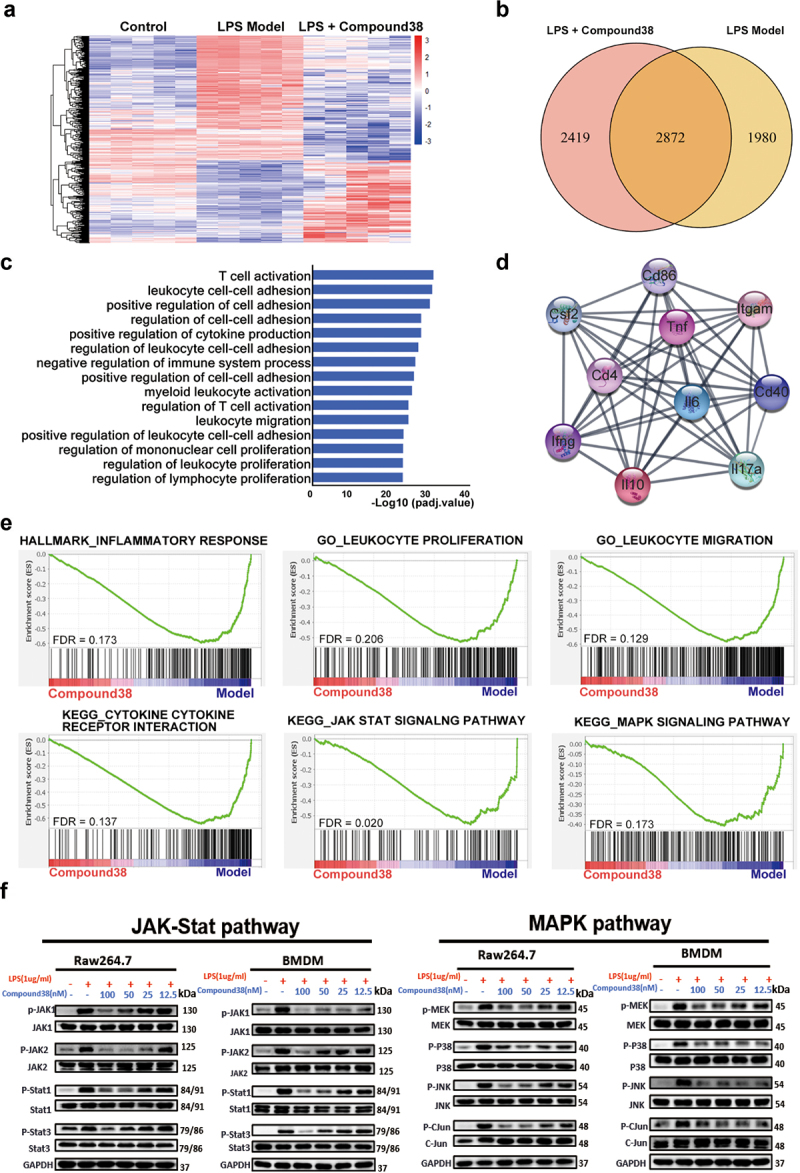
Compound38 inhibits pro-inflammatory gene expression and blocks the inflammatory pathway.

To further investigate the potential mechanism underlying the remarkable anti-inflammatory effect of compound 38, we adopted RNA-seq analysis to obtain a high-resolution picture of the dynamic and complex transcriptomes in the liver tissues from different treatment groups. The results of unsupervised hierarchical clustering revealed that the clustering gene expression patterns between the control group and the LPS/GalN-exposed group were quite different, whereas the clustering patterns of the compound 38-treated group were similar to those of the control group, indicating that a broad set of pathologic genes was suppressed (Figure 3a). Variations in 4852 genes were stimulated by LPS/GalN exposure, most of which were related to inflammatory cytokines. As shown in the Venn diagram in Figure 3b, approximately 60% (2872) of the changes in genes affected by LPS/GalN exposure were reversed in the compound 38-treated group.

(a–d) Total RNA for RNA-seq analysis was extracted from the liver tissues of mice in the control group (n = 5), LPS/GalN-exposure group (n = 6), and compound 38-treated group (n = 5). (a) Heatmap showing the different clustering gene expression patterns among the control, LPS/GalN exposure, and compound 38-treated groups; differentially expressed genes were filtered for an adjusted P value < 0.05 and absolute value of log_2_ fold-change > 1. (b) The overlap between the gene set upregulated by LPS/GalN and the gene set subsequently downregulated by compound 38 *in vivo* presented as a Venn diagram. (c) Gene-annotation enrichment analysis performed using differentially expressed genes between the LPS/GalN model group and the compound 38-treated group, revealing that the differentially expressed genes were enriched in pathways associated with the inflammatory response. (d) Visualization of the top 10 hub genes and their PPI network. (e) GSEA results revealing the biological processes affected by compound 38. (f) Western blot analysis validating that compound 38 regulates the JAK-STAT and MAPK signaling pathways in Raw264.7 and primary BMDM cells. *P < 0.05, **P < 0.01, ***P < 0.001, ****P < 0.0001 compared to LPS/GalN-stimulated mice. #P < 0.05, ##P < 0.01, ###P < 0.001, ####P < 0.0001 compared to the control group.

Gene-annotation enrichment analysis showed that differentially expressed genes from the LPS/GalN-exposed group and compound 38-treated group were enriched in the biological processes related to the proliferation and migration of immune cells, which play a critical role in the inflammatory response (Figure 3c). The genes from the top 10 biological processes affected by compound 38 were mapped to the PPI network. The hub genes in the PPI network (i.e., those with the top 10% connectivity) were associated with inflammatory cytokines, such as *Il6, Il10, Il17a, Tnf, Ifng*, and *Csf2*. The other hub genes, *Cd4, Cd40, Cd86*, and *Itgam*, have also been reported to be closely related to the inflammatory response (Figure 3d). The strong inflammatory response caused by high expression of these hub genes gave rise to the pathological phenotype of acute liver injury; nonetheless, compound 38 strongly suppressed the injury caused by these inflammatory cytokines.

GSEA was further adopted to ascertain whether the specific gene sets of inflammation-related pathways or biological processes differed significantly between the two groups. The expression levels of most genes from the hallmark gene set of inflammatory response were remarkably decreased, reflecting the reduction of inflammation following compound 38 treatment. The enrichment plot also showed that compound 38 effectively suppressed the proliferation and migration of leukocytes, which are specific biological processes in the inflammatory response. In addition, the significantly enriched pathways related to inflammatory cytokines, including the mitogen-activated protein kinase (MAPK) and Janus kinase-signal transducer and activator of transcription (JAK-STAT) signaling pathways, were distinctly diminished in the compound 38-treated group. Western blot analysis confirmed these changes in the key proteins involved in the MAPK and JAK-STAT signaling pathways in both primary BMDM and Raw264.7 cells. Thus, sufficient and solid evidence with RNA-seq and western blots elucidated the common mechanism contributing to the anti-inflammatory effects of compound 38.

### Compound 38 ameliorated CCl_4_-induced liver fibrosis

#### Compound 38 administration alleviated HSC activation

Liver fibrosis, characterized by excessive ECM deposition, is a dynamic process involving acute or chronic etiological injury. During liver injury, HSCs, which maintain a quiescent, non-proliferative cell phenotype in the normal liver, are activated and transdifferentiate into myofibroblasts producing ECM. The activation of HSCs has been considered as the critical trigger of liver fibrosis in humans and in experimental liver injury models [7]. Thus, inhibition of HSC activation is the key to the effective treatment of fibrotic liver diseases.

**Figure 4.**
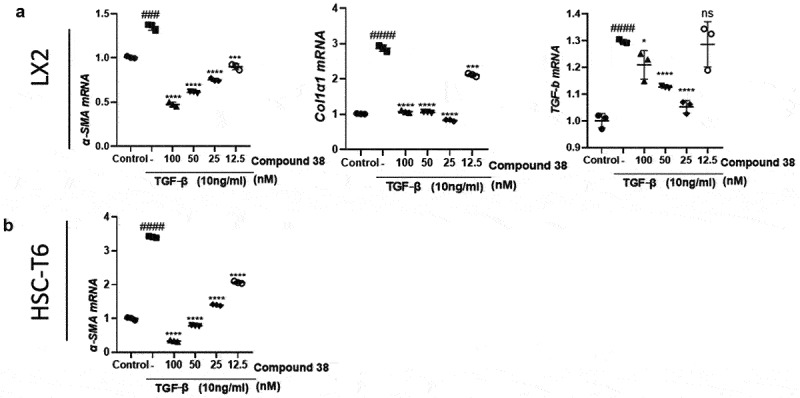
Compound 38 treatment inhibited hepatic stellate cell (HSC) activation.

We first explored the anti-fibrotic effect of compound 38 in different HSC cell lines (human LX2 cells and rat HSC-T6 cells). TGF-β stimulation elevated the mRNA levels of fibrotic genes, including *α-SMA, COL1A1*, and *TGF-β*, which were inhibited by compound 38 administration in a concentration-dependent manner (Figure 4a, b)

(a) The human HSC cell line LX-2 was starved in serum-free medium for 24 h and then treated with compound 38 and TGF-β1 (10 ng/mL) for 24 h, after which *α-SMA, COL1A1*, and *TGF-β* mRNAs were quantified by RT-qPCR analysis. (b) The rat HSC cell line HSC-T6 was cultured in serum-free medium for 24 h and then treated with compound 38 and TGF-β1 (10 ng/mL) for 24 h, after which *α-Sma* mRNAs were quantified by RT-qPCR analysis. Data represent the mean ± SD. *P < 0.05, **P < 0.01, ***P < 0.001,****P < 0.0001 compared to the TGF-β1-stimulated group. #P < 0.05, ##P < 0.01, ###P < 0.001, ####P < 0.0001 compared to the control group.

#### Compound 38 administration protects against liver fibrosis in vivo

**Figure 5.**
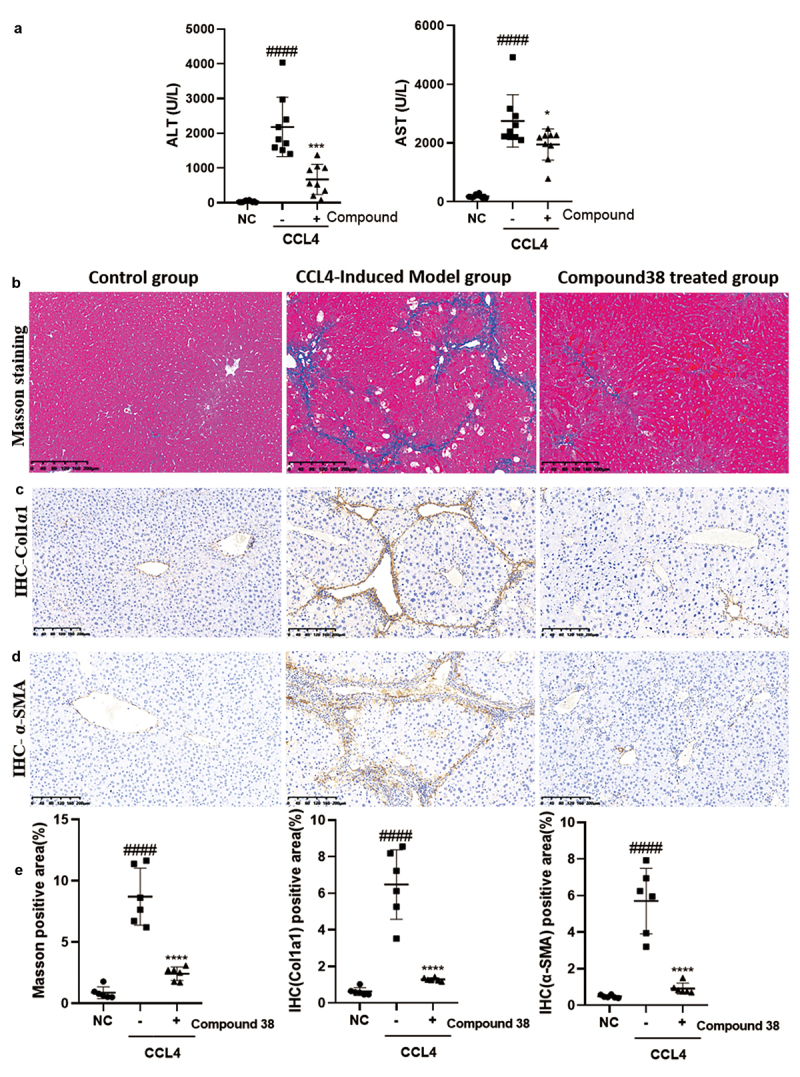
Compound 38 treatment reduces liver inflammation and fibrosis.

Chronic persistent inflammation causes liver fibrosis. Based on the anti-inflammatory and anti-fibrotic functions of compound 38 *in vitro*, we further explored these effects *in vivo* in the CCl_4_-induced liver fibrosis mouse model. Treatment of CCl_4_ (1 mL/kg, i.p.) for 8 weeks resulted in a significant increase in serum transaminase levels, whereas compound 38 treatment decreased the degree of CCl_4_-induced liver injury (Figure 5a).

Mice received intraperitoneal injections of CCl_4_ (10% CCl_4_ dissolved in olive oil at a dose of 1 mL/kg body weight) twice a week. (a) Serum ALT and AST levels in mice from the normal control (NC; n = 11), CCl_4_-induced liver fibrosis model (n = 9), and model with compound 38 treatment (n = 9) groups. (b) Representative images of Masson staining from the mouse liver tissues of the indicated groups. (c and d) Representative images of immunohistochemical staining for COL1α1 (c) and α-SMA (d) in liver sections. (e) Quantification of different stainings (n = 10 images per group from the NC, CCl_4_, and CCl_4_ + compound 38 groups; six samples per group). Data represent the mean ± SD. *P < 0.05, **P < 0.01, ***P < 0.001, ****P < 0.0001 compared to CCl_4_-induced mice. #P < 0.05, ##P < 0.01, ###P < 0.001, ####P < 0.0001 compared to the control group.

α-SMA and COL1α1 are indicators of HSC activation following liver injury, and ECM proteins are abundant in fibrotic liver tissues **[**52–54**]**. To further confirm that the model was successfully established and evaluate the anti-fibrotic effect of compound 38 *in vivo*, we performed Masson staining and immunohistochemical staining for COL1α1 (Figure 5c) and α-SMA (Figure 5d) in the liver sections of mice. The CCl_4_ treatment facilitated perisinusoidal or pericellular fibrosis, and caused distortion of the liver parenchyma, whereas compound 38 treatment reversed this disorder to a significant extent, decreased tissue fibrosis (Figure 5b), and reduced the deposition of the marker proteins COL1α1 (Figure 5c) and α-SMA (Figure 5d). Semi-quantitative analysis showed significant differences in staining among the control group, CCl_4_-induced group, and compound 38-treated group (Figure 5e). These data indicated that compound 38 has the ability to suppress the progression of liver fibrosis *in vivo*.

### Compound 38 inhibited the TGFβ/SMAD and Wnt/β-catenin signaling pathways to inactivate HSCs

**Figure 6.**
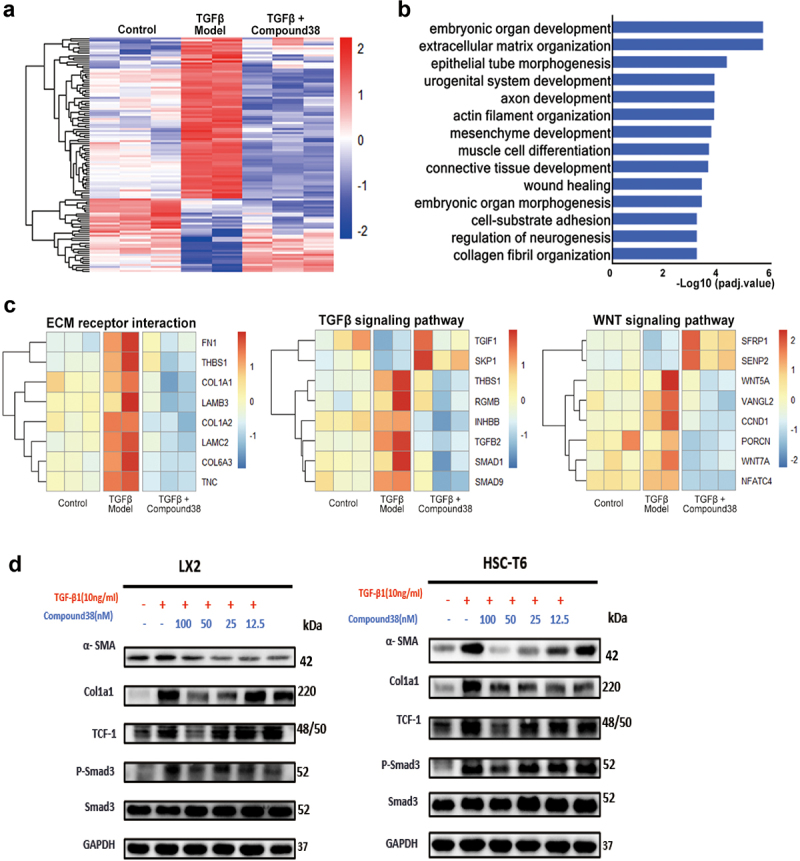
Compound 38 blocks fibrosis-related pathway activation in LX2 cells.

RNA-seq analysis was used to provide more comprehensive insight into the mechanism by which compound 38 distinctly reversed the pathological phenotype of liver fibrosis. The heatmap revealed that the clustering gene expression patterns of the TGF-β-induced group were different from those of the control group, which were reversed in the compound 38-treated group, with a major effect at 12.5 nM (Figure 6a). GSEA demonstrated that the differentially expressed genes are involved in many biological processes related to fibrosis such as ECM organization, mesenchymal development, connective tissue development, wound healing, and collagen fibril organization (Figure 6b).

(a–c) Total RNA for RNA-seq analysis was extracted from LX2 cells in the control group (n = 3), TGF-β exposure group (n = 2), and compound 38-treated group (50 nM, n = 3). (a) Heatmap showing the different clustering gene expression patterns among the control, TGF-β1 exposure, and compound 38-treated groups; differentially expressed genes were filtered for an adjusted P value < 0.05 and absolute value of fold-change > 1.5. (b) Gene-annotation enrichment analysis performed on differentially expressed genes between the TGF-β exposure group and the compound 38-treated group. (c) Heatmap revealing the key genes of biological processes and pathways affected by compound 38. (d) Western blot analysis to validate activation of the TGF-β/SMAD and Wnt/β-catenin signaling pathways in the human HSC cell line LX-2 and the rat HSC cell line HSC-T6. *P < 0.05, **P < 0.01, ***P < 0.001, ****P < 0.0001 compared to the TGF-β-induced group. #P < 0.05, ##P < 0.01, ###P < 0.001, ####P < 0.0001 compared to the control group.

Moreover, the heatmaps displayed the expression level of typical genes in ECM receptor interaction, and the TGF-β/SMAD and Wnt/β-catenin signaling pathways (Figure 6c). The genes shown in the heatmap of ECM receptor interaction are primary components of the ECM, which were significantly reduced after administration of compound 38, reflecting that the phenotypes of liver fibrosis were effectively improved. In addition, among the significant differentially expressed genes in the compound 38-treated group, *TGFB2* and *SMAD1/9*, key effectors in the TGF-β signaling pathway, were remarkably suppressed; *TGIF1* and *SKP1*, the negative regulators of the TGF-β signaling pathway, were upregulated; and the positive regulators *THBS1* and *INHBB* were downregulated. Similarly, the expression levels of critical genes in the Wnt signaling pathway *WNT5A/7A* and *CCND1* were markedly reduced, the negative regulators of Wnt signaling *SFRP1* and *SENP2* were upregulated, and the positive regulator *VANGL2* was downregulated in the compound 38-treated group. These results demonstrated that compound 38 inactivates HSCs through inhibiting the TGF-β/SMAD and Wnt/β-catenin signaling pathways. As validated by western blotting, the protein levels of α-SMA, TCF-1, COL1α1, and phosphorylated-SMAD3 were decreased by compound 38 in a concentration-dependent manner, revealing that the pathogenicity of the fibrotic phenotype was significantly attenuated in the LX2 and HSC-T6 cell lines (Figure 6d).

## Discussion

Liver fibrosis, triggered by injurious inflammation, is a common outcome of various liver diseases, including chronic virus infection, alcohol abuse, and nonalcoholic fatty liver, and different types of parenchymal and mesenchymal cells have recently been found to underlie liver fibrosis by means of their pathological actions and interactions [6,55]. Because of these complicated underlying mechanisms, there is still no effective clinical treatment for liver fibrosis, especially by drug administration.

Pathophysiologically, macrophages are pivotal in maintaining tissue homeostasis and mediating injury-based immune responses due to a variety of factors [51,56]. Kupffer cells, as a liver-resident macrophage population, have been widely accepted to be activated following liver injury, leading to the release of inflammatory cytokines [56–58]. Inflammatory filtration occurs in the hepatic lobula. In our experiments, LPS-induced acute hepatic injury increased the level of the Kupffer cell-specific signal F4/80 throughout the liver tissue. When compared with those of the compound 38-treated group, mice in the model group exhibited significantly upregulated hepatic expression of *Tnf-α, Il-6*, and *Il-1β* mRNAs. Immunohistochemical staining for LY-6 G also revealed the hepatic enrichment of neutrophils, which were likely recruited by inflammatory cytokines. Overall, our data suggest that liver inflammation is induced by LPS-dependent liver injury and related Kupffer cell activation, and that administration of compound 38 dramatically reduced the number of activated Kupffer cells after LPS stimulation and successively decreased both inflammatory cytokine levels and neutrophil infiltration. These pharmacological actions further protected mice from hepatocyte impairment and death within one month.

**Figure 7.**
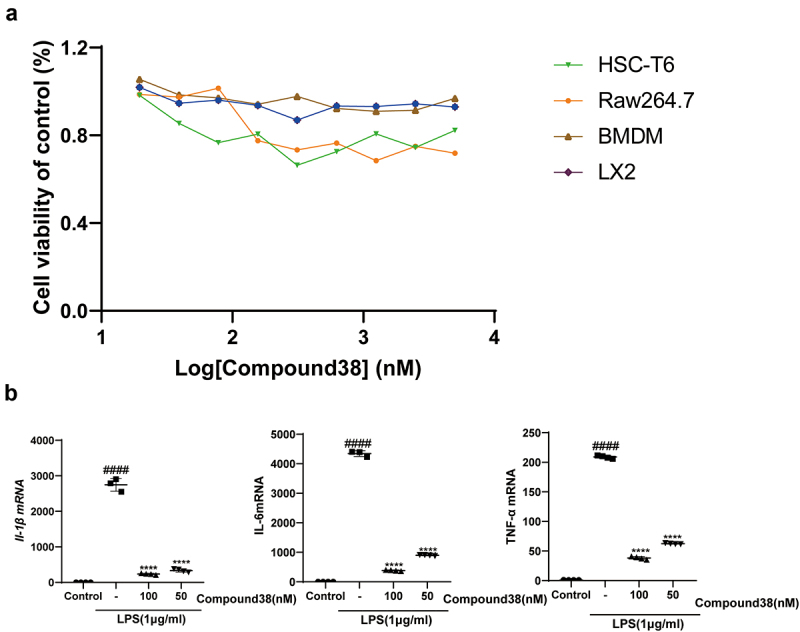
(a) The viability of four kinds of cells was assessed using the Cell Titer-Glo assay after 24-h administration by compound 38. (b) Four-hour administration with the selective BET inhibitor compound 38 downregulated the mRNA levels of Il-1β, Il-6, and Tnf-α in mouse Kupffer cells.*P < 0.05, **P < 0.01, ***P < 0.001, ****P < 0.0001 compared to the TGF-β-induced group. #P < 0.05, ##P < 0.01, ###P < 0.001, ####P < 0.0001 compared to the control group.

BET bromodomain inhibition contributes to the suppression of profibrotic transcriptional networks and innate inflammatory responses [23,25,59–64]. Analysis of the mechanisms underlying the protective effects of treatment of the selective BET bromodomain inhibitor compound 38 *in vivo* and *in vitro* showed that the compound has a significant impact on many inflammation-related signaling pathways such as the JAK-STAT and MAPK signaling pathways. PPI network analysis revealed the inflammation-promoting effects with a focus on the cytokines *IL6, IL10, IL17a, Tnf, Ifng*, and *Csf2*, and other hub genes *Cd4, Cd40, Cd86*, and *Itgam*. Downregulated expression of key genes associated with the JAK-STAT, ERK, P38, and JNK signaling pathways was also found in macrophage cell lines at both the transcription and translation levels. Thus, the selective BET bromodomain inhibitor compound 38 is indicated to play an anti-inflammatory role via the inactivation of Kupffer cells, at least partly by inhibition of the JAK-STAT and MAPK signaling pathways (Figure 7a).

Following inflammatory stimuli, HSCs in the sinusoid space of the liver undergo transdifferentiation from a lipid-containing phenotype to a fibroblast-like phenotype (myofibroblasts), which is otherwise known as HSC activation [7]. This activation results in liver fibrosis/cirrhosis, causing a disbalance of ECM synthesis and degradation, mainly depending on the profibrotic niche of the ECM (e.g., collagens, Hyaluronic acid, and laminin,), cytokines, and chemical media [10,55,65–67]. Activation of the Wnt/β-catenin and TGF-β/SMAD pathways underlying HSC activation is responsible for liver fibrosis [68]. After the injurious stimulation and increase in proinflammatory cytokines, our experiments verified the high activity of the TGF-β/SMAD and Wnt/β-catenin signaling pathways, and significant upregulation of α-SMA, TGF-β, and COL1α1 expression, which are specific to HSC activation. Excessive deposition of the ECM resulted in the formation of fibrotic septa connecting the central veins and portal tracts, along with pseudo-lobules. However, treatment with the selective BET bromodomain inhibitor compound 38 decreased the expression levels of *TGF-β, COL1A1*, and *α-SMA* mRNAs. The inhibition of inflammatory activity was accompanied by a dose-dependent decrease in markers of activated HSCs and fibrogenesis. Consistent with these findings, mice in the compound 38-treated group exhibited less fibrotic septa and a lower fibrotic area as compared with those in the model group. RNA-seq, bioinformatics analysis, and western blotting suggested that the anti-fibrotic effect of compound 38 was involved in the inactivation of the TGFβ/SMAD and Wnt/β-catenin signaling pathways.

In conclusion, compound 38, a selective BET bromodomain inhibitor, inactivates the JAK-STAT and MAPK signaling pathways to inhibit the Kupffer-based inflammatory response. Moreover, compound 38 abolishes HSC activation by inactivating the Wnt/β-catenin and TGF-β/SMAD signaling pathways. These simultaneous pharmacological actions effectively treated injurious inflammation and fibrogenesis to mitigate liver fibrosis in mice, which qualifies compound 38 as a strong candidate for clinical therapy. However, given the difference between human disease and animal models, more research is needed to translate the current results for the clinical treatment of human diseases.

## Supplementary Material

Supplemental Material
