# Supplementary material for: Selective bromodomain and extra-terminal bromodomain inhibitor inactivates macrophages and hepatic stellate cells to inhibit liver inflammation and fibrosis
Source: Bioengineered. 2022 May 1;13(4):10914–30. doi: 10.1080/21655979.2022.2066756 (PMC9278415; doi:10.1080/21655979.2022.2066756)
Supplement: Supplemental Material [file KBIE_A_2066756_SM7231.zip › supplementary/Table S1.docx]

Table S1. Primers used for real-time PCR studies

| Genes |  | Oligonucleotides (5’-3’) |
| --- | --- | --- |
| *Gapdh* | Forward | AGGTCGGTGTGAACGGATTTG |
|  | Reverse | TGTAGACCATGTAGTTGAGGTCA |
| *Il1β* | Forward | GAAATGCCACCTTTTGACAGTG |
|  | Reverse | TGGATGCTCTCATCAGGACAG |
| *Il6* | Forward | CTGCAAGAGACTTCCATCCAG |
|  | Reverse | AGTGGTATAGACAGGTCTGTTGG |
| *Tnfα* | Forward | CGATCACCCCGAAGTTCAGTAG |
|  | Reverse | CAGGCGGTGCCTATGTCTC |
| *β-ACTIN* | Forward | GCTGAAGTATCCGATAGAACACG |
|  | Reverse | GGTCTCAAACATAATCTGGGTCA |
| *α-SMA* | Forward | CGTGGCTATTCCTTCGTTAC |
|  | Reverse | TGCCAGCAGACTCCATCC |
| *COL1A1* | Forward | GTGCGATGACGTGATCTGTGA |
|  | Reverse | CGGTGGTTTCTTGGTCGGT |
| *TGFβ* | Forward | GGCCAGATCCTGTCCAAGC |
|  | Reverse | GTGGGTTTCCACCATTAGCAC |
| *GAPDH* | Forward | AGGTCGGTGTGAACGGATTTG |
|  | Reverse | GGGGTCGTTGATGGCAACA |
| *α-sma* | Forward | GAGGGATCCTGACCCTGAAG |
|  | Reverse | CCACGCGAAGCTCGTTATAG |
| *β-actin* | Forward | GAGCGCAAGTACTCTGTGTG |
|  | Reverse | CCTGCTTGCTGATCCACATC |
