# Supplementary material for: Selective bromodomain and extra-terminal bromodomain inhibitor inactivates macrophages and hepatic stellate cells to inhibit liver inflammation and fibrosis
Source: Bioengineered. 2022 May 1;13(4):10914–30. doi: 10.1080/21655979.2022.2066756 (PMC9278415; doi:10.1080/21655979.2022.2066756)
Supplement: Supplemental Material [file KBIE_A_2066756_SM7231.zip › supplementary/Table S2.docx]

Table S2. Antibodies used for Western Blot studies

| JNK | Cell Signaling Technology | Cat# 9252 |
| --- | --- | --- |
| p-JNK | Cell Signaling Technology | Cat# 4668 |
| P38 | Cell Signaling Technology | Cat# 8690 |
| P-P38 | Cell Signaling Technology | Cat# 9216 |
| MEK1/2 | Cell Signaling Technology | Cat# 4694 |
| P-MEK1/2 | Cell Signaling Technology | Cat# 2338 |
| c-Jun | Cell Signaling Technology | Cat# 9165 |
| P-c-Jun | Cell Signaling Technology | Cat# 3270 |
| JAK1 | Cell Signaling Technology | Cat#50996 |
| P-JAK1 | Cell Signaling Technology | Cat#74129 |
| JAK2 | Cell Signaling Technology | Cat#3230 |
| P-JAK2 | Cell Signaling Technology | Cat#4406 |
| Stat1 | Cell Signaling Technology | Cat#14994 |
| P-Stat1 | Cell Signaling Technology | Cat#7649 |
| Stat3 | Cell Signaling Technology | Cat#12640 |
| P-Stat3 | Cell Signaling Technology | Cat#9145 |
